# NLRP3 Inflammasome Activation Expands the Immunosuppressive Myeloid Stroma and Antagonizes the Therapeutic Benefit of STING Activation in Glioblastoma

**DOI:** 10.1158/2767-9764.CRC-23-0189

**Published:** 2025-06-13

**Authors:** Spencer T. Lea, Chao-Hsien Chen, Jun Wei, Ivana William, Inés Lopez Del Castillo, Michael A. Curran

**Affiliations:** 1 Department of Immunology, The University of Texas MD Anderson Cancer Center, Houston, Texas.; 2 The University of Texas MD Anderson Cancer Center UTHealth Graduate School of Biomedical Sciences, Houston, Texas.; 3 Department of Cell Biology, University of Málaga, Málaga, Spain.

## Abstract

Glioblastoma (GBM) is the most common and deadly primary brain malignancy and is clinically refractory to immunotherapy. Active NLRP3 inflammasome signaling and IL-1β secretion have been observed in GBM, and NLRP3-driven myeloid-derived suppressor cell (MDSC) recruitment can mediate cancer immune evasion. Agonists of the cytosolic double-stranded DNA–sensing stimulator of IFN gene (STING) pathway can mediate proinflammatory conversion of cancer MDSCs; however, secretion of the NLRP3 products IL-1β and IL-18 has also been observed in certain myeloid populations following STING activation. In this study, we aimed to determine both the potential mechanistic synergy between STING and NLRP3 agonists, and the effects of this innate immune combination on the GBM tumor immune landscape. We find that STING activation does not prime pro–IL-1β expression for activated NLRP3 inflammasome secretion. In subcutaneous GL261 GBM, we show that NLRP3 activation expands the immunosuppressive myeloid stroma primarily via granulocytic MDSC recruitment and antagonizes the benefit of STING activation. In brain GL261, we find that NLRP3 activation expands granulocytic MDSCs but does not antagonize the therapeutic benefit of STING activation. Finally, we report that mesenchymal subtype GBM tumors have elevated neutrophil, IL-1β, and *NLRP3* gene expression, a setting where our data suggest that NLRP3 activation could counteract STING agonists.

**Significance:**

NLRP3 inflammasome signaling, which suppresses antitumor immunity in some cancers, has been observed in GBM tissues. NLRP3 activation in GBM induces granulocyte-dependent tumor immunosuppression and antagonizes the therapeutic efficacy of STING activation.

## Introduction

Glioblastoma (GBM) is the most common and aggressive adult primary brain malignancy, with a dismal median overall survival of only 15 months after diagnosis (1). Despite significant interest and preclinical effort in developing immunotherapies for GBM, it is clinically refractory to T-cell immune checkpoint blockade (2, 3). This is in part due to its dense immunosuppressive myeloid stroma, consisting largely of bone marrow–derived macrophages (BMDM), brain-resident microglia, and myeloid-derived suppressor cells (MDSC; ref. 4, 5). These immunosuppressive populations are sensitive to agonists of the double-stranded DNA–sensing stimulator of IFN gene (STING) pathway, and STING agonists in GBM and other cancers have demonstrated curative responses and proinflammatory conversion of infiltrating myeloid populations (6–11). Studies have shown that STING activation–induced tumor rejection is largely driven by the IRF3-induced type 1 IFN response (12, 13). However, the STING also signals through the NF-κB transcription factor, and STING agonists have been shown to induce secretion of several cytokines including IL-1β and IL-18 (7, 14, 15). Notably, IL-1β and IL-18 are the primary cytokines secreted upon NLRP3 inflammasome activation (16).

Inflammasomes including NLRP3 form in the cytosol as a response to damage- and pathogen-associated molecular patterns, and the fully activated NLRP3 complex is responsible for cleaving proform IL-1β and IL-18 into fully mature cytokines for secretion (16). NLRP3 inflammasome activation is proposed to occur in two steps. First, a priming stimulus such as Toll-like receptor (TLR) activation activates NF-κB, which stimulates transcription of NLRP3 and pro–IL-1β proteins. Second, an activating stimulus such as sensing of extracellular ATP leads to NLRP3 inflammasome complex formation and caspase-1 cleavage of pro–IL-1β and pro–IL-18 (16, 17).

Despite the reported proinflammatory functions of IL-1β in infection, NLRP3 activation can also contribute to tumor pathogenesis and has proved to be detrimental to antitumor immunity in several cancers including melanoma (18, 19). Clinically, a retrospective analysis of a phase 3 study of the IL-1β inhibitor canakinumab in patients with atherosclerosis revealed significantly lower lung cancer incidence and mortality in patients receiving IL-1β inhibition (20). Although it has been shown that GBM can aberrantly express IL-1β, and NLRP3 inhibition can reduce GBM tumor growth (21–24), the link between NLRP3 activation and antitumor immunity has not been studied in GBM.

Studies have linked the cyclic GMP–AMP (cGAMP) synthase (cGAS)–produced dinucleotide 2′3′cGAMP to inflammasome activation following lipopolysaccharide (LPS) priming in an NLRP3-dependent manner and have also suggested that 2′3′cGAMP can weakly prime IL-1β expression, albeit at levels about 10- to 20-fold lower than observed with LPS stimulation (15). Due to the significant preclinical and clinical interest in synthetic STING agonism in GBM, and the potential immunosuppressive effects of NLRP3 activation, we sought to determine whether synthetic agonist-induced STING NF-κB signaling can prime pro–IL-1β expression for inflammasome processing. We then sought to determine whether NLRP3 activation in GBM drives expansion of tumor-infiltrating immunosuppressive populations, and, if so, whether STING activation remains capable of proinflammatory repolarization of an NLRP3-activated GBM tumor microenvironment. We report that synthetic STING activation does not prime the NLRP3 inflammasome for IL-1β secretion *in vitro*, and NLRP3 activation expands the immunosuppressive GBM myeloid infiltrate and antagonizes the therapeutic benefit of STING activation *in vivo* in peripheral subcutaneous GL261 GBM tumors but not in intracranial GL261 GBM tumors. Using high dimensional flow cytometry, we show that NLRP3 activation reverses STING agonist-driven pro-inflammatory effects in the subcutaneous GBM tumor microenvironment including increased infiltration of granzyme^+^ NK cells, improved therapeutic ratios of CD8 T effector (Teff) cells to CD4 Tregs, and M2 to M1-like proinflammatory conversion of infiltrating monocyte-derived populations.

We have previously found that the majority of the human glioma-associated myeloid compartment is comprised of microglia, tumor-associated macrophages (TAM), and monocytic MDSCs (CD14^+^) with a minimal granulocytic MDSC contribution (5). Our group has previously shown that when implanted in the central nervous system (CNS), baseline GL261 is similarly poorly infiltrated by neutrophils (25). In this setting, we find that NLRP3 activation neither confers therapeutic benefit as a monotherapy nor antagonizes the therapeutic benefit of STING activation when used in combination. Despite the lack of therapeutic antagonism, we similarly find using high-dimensional flow cytometry of GL261 tumors implanted in the CNS that NLRP3 activation expands the immunosuppressive myeloid infiltrate.

Finally, using RNA sequencing (RNA-seq) transcriptomic profiling data from The Cancer Genome Atlas (TCGA) GBM project data, we show that patients with low neutrophil gene signature expression have extended survival, and the mesenchymal molecular GBM subtype has significantly elevated expression of neutrophil gene signatures, IL-1β and NLRP3. Our findings suggest that neutrophil-rich settings like mesenchymal subtype tumors may more closely resemble the flank GL261 setting and could be adversely affected by NLRP3 agonist therapy.

## Materials and Methods

### Mice

Male C57BL/6 mice (RRID: IMSR_JAX:000664) were purchased from The Jackson Laboratory and were between 8 and 10 weeks of age. All mouse studies were performed in compliance with the approved protocols 00001378-RN01 and 00001378-RN02 by the Institutional Animal Care and Use Committee at MD Anderson Cancer Center.

### Cell lines

The GL261 cell line (a gift of the Amy Heimberger Lab; RRID: CVCL_Y003) was maintained in complete DMEM supplemented with 10% FBS (Gibco), penicillin–streptomycin (Gibco), and 2 mmol/L L-glutamine. The THP1 Dual reporter cell line was obtained from InvivoGen (RRID: CVCL_X599) and cultured according to the manufacturer’s instructions in complete RPMI (cRPMI) medium supplemented with 10% FBS (Gibco), penicillin–streptomycin (Gibco), and 2 mmol/L L-glutamine and blasticidin (30 μg/mL) and Zeocin (100 μg/mL) for reporter selection. *Mycoplasma* testing and cell authentication of the noncommercial cell lines was performed by the MD Anderson Cytogenetics and Cell Authentication Core.

### Small-molecule agonists and inhibitors

Cyclic di-GMP (cdGMP), LPS, nigericin, and MCC950 were purchased from InvivoGen and reconstituted as directed. ML-RR-S2-CDA (MLRR) was synthesized by Wuxi and reconstituted in water.

### THP1-Dual reporter assays

THP1-Dual reporter assays were performed according to InvivoGen instructions. Cells were harvested, washed, and resuspended at 2.0 × 10^5^ cells/well in a flat bottom 96-well plate. THP1 cells were incubated overnight with MLRR or LPS at indicated concentrations, then supernatant harvested, and NF-κB induction measured using QUANTI-Blue solution (InvivoGen) as indicated. For IL-1β ELISA, THP1 cells were resuspended at 2.0 × 10^4^ in a flat-bottom 96-well plate. THP1 cells were incubated overnight in LPS or MLRR as indicated (priming) and then treated with an additional 20 μL of MCC950 and/or nigericin as indicated for 2 hours (activation). The supernatant was then harvested and the IL-1β concentration measured using the Invitrogen IL-1β Human ELISA Kit.

### BMDM assays

Bone marrow was isolated from male C57BL/6 mice via aspiration of the femur. Red blood cell lysis was performed using red blood cell lysis buffer (Sigma-Aldrich), and cells were plated in one T75 tissue culture (TC)-treated flask per femur in 25 mL of cRPMI medium plus 50 nmol/L macrophage colony-stimulating factor (M-CSF). The following day, media including floating cells was transferred to one 15-cm TC-treated plate per femur, and cells incubated for 6 days. Macrophages were harvested, washed, and resuspended to 3.0 × 10^6^ cells/mL and incubated overnight at 3.0 × 10^5^ cells/well in a flat bottom non-TC–treated 96-well plate with MLRR or LPS. Following overnight incubation, macrophages were harvested and processed for RNA isolation and flow cytometry. Intracellular pro–IL-1β expression was analyzed by flow cytometry. RNA was isolated (Qiagen RNeasy kit), and IL-1β relative gene expression to untreated macrophages analyzed via qPCR (TaqMan; Thermo Fisher Scientific). For IL-1β ELISA, BMDM cells were incubated overnight in LPS or MLRR as indicated (priming) and then treated with an additional 20 μL MCC950 and/or nigericin as indicated for 2 hours (activation). The supernatant was then harvested and the IL-1β concentration measured via murine IL-1β ELISA (R&D Systems).

### GL261 implantation and treatments

GL261 cells were harvested with 0.05% trypsin, washed with PBS, filtered through a 70-micron cell strainer, and counted. Cells were resuspended in 70% cold PBS plus 30% Matrigel for implantation of 1.0 × 10^6^ cells/mouse in 100 μL/mouse subcutaneously in the left flank of C57BL/6 mice. Palpable tumors were treated intratumorally with 50 μL of cdGMP and/or nigericin as indicated on days 10, 14, and 18. Tumor volumes were measured every 3 to 4 days until reaching a volume of 1,000 mm^3^ for growth and survival analysis, at which time mice were humanely euthanized. For tumor immune infiltrate analysis, mice were euthanized 48 hours following the final day 18 treatment and processed for flow cytometry analysis. For intracranial GBM studies, GL261 cells were resuspended in 100% cold PBS for implantation of 5.0 × 10^4^ cells in the right striatum via a guide bolt system. Intracranial tumors were then treated on day 7 with 5 μL of cdGMP and/or nigericin intratumorally through the previously implanted guide bolts, and mice were monitored for survival. Moribund mice displaying neurologic symptoms were humanely euthanized.

### Flow cytometry analysis of GL261 immune infiltrates

GL261 tumors were implanted and treated subcutaneously or intracranially as described. Following euthanasia, subcutaneous tumors or tumor-bearing brain hemispheres and naïve spleen and brain controls were harvested, weighed, and diced into 70-micron filters in 6 cm plates containing tumor digestion media [serum-free RPMI medium containing collagenase (1 mg/mL, Sigma-Aldrich) and DNase (160 μg/mL, Roche)] for 30 minutes at 37°C. Tumors were then mashed through filters and single-cell suspensions washed and counted. Live immune cells harvested from subcutaneous tumors were purified by Ficoll gradient centrifugation (Histopaque-1119, Sigma-Aldrich). Myelin was removed and live immune cells enriched from intracranial tumors using Percoll density gradient centrifugation (Sigma-Aldrich). Samples were transferred to a round bottom 96-well plate for flow cytometry staining, then stained with Near-IR fixable viability dye (Invitrogen), washed, and fixed overnight with eBioscience fixation/permeabilization kit (Thermo Fisher Scientific). Samples were stained with fluorescently labeled antibodies for flow cytometry analysis.

### Flow cytometry staining

Samples were washed in U-bottom 96-well plates with FACS buffer and then stained for 10 minutes at 4°C with Near-IR viability dye (Invitrogen). Samples were again washed with FACS buffer and then fixed/permeabilized with eBioscience fixation/permeabilization kit (Thermo Fisher Scientific). Cells were washed in permeabilization buffer (Thermo Fisher Scientific) and incubated for 15 minutes at 4°C with anti-mouse CD32/CD16 Fc-block antibody (Leinco; RRID: AB_2829545), followed by incubation for 1 hour at 4°C with fluorescently labeled antibodies. Following staining, samples were washed and analyzed using a BD LSRFortessa X-30 prototype flow cytometer (subcutaneous tumors) or Cytek Aurora spectral cytometer (intracranial tumors). Flow cytometry data were analyzed using the FlowJo application (BD Biosciences; RRID: SCR_008520).

### Human macrophage generation

Healthy donor buffy coats were obtained from the University of Texas MD Anderson Cancer Center Blood Bank. Peripheral blood mononuclear cells were isolated by Ficoll gradient centrifugation (Histopaque-1077; Sigma-Aldrich), and CD14^+^ monocytes were isolated by negative selection using the Miltenyi Classical Monocyte Isolation Kit. Monocytes were then differentiated into M2c macrophages. In short, monocytes were plated in T25 flasks (1 × 10^6^ cells/mL) in RPMI medium containing 20% FBS and 100 ng/mL recombinant human M-CSF for 6 days, with a media refresh on day 4. On day 6, media was replaced, with the addition of recombinant human TGF-β (10 ng/mL) and IL-10 (10 ng/mL). On day 8, adherent cells were washed with PBS and cultured with cRPMI + 100 ng/mL M-CSF + 10 µg/mL indicated cyclic dinucleotide for repolarization over 72 hours. Adherent macrophages were harvested with Detachin cell detachment solution (Genlantis) for downstream applications. All recombinant human cytokines were purchased from PeproTech and reconstituted according to the manufacturer’s instructions.

### Luminex sample preparation

Repolarized human M2c macrophages were generated as described. Supernatants from repolarized cultures were isolated, spun to remove contaminating cells, and then frozen at −80°C. All harvested supernatants were thawed on ice and were analyzed with the Cytokine & Chemokine 36-Plex Mouse ProcartaPlex or Cytokine/Chemokine/Growth Factor 45-Plex Human ProcartaPlex Panels (Invitrogen/Thermo Fisher Scientific) using a Luminex MAGPIX machine according to the manufacturer’s instructions.

### Analysis of patients in TCGA GBM cohort

Transcriptome bulk RNA-seq data and accompanying patient clinical data from The Cancer Genome Atlas (RRID: SCR_003193) were downloaded in R software (RRID: SCR_001905) v4.3.2. Sequencing data was normalized using the Bioconductor limma package and voom normalization function. Neutrophil gene signatures were defined as combined expression of S100A8, S100A9, and ITGAX in bulk RNA-seq (26). Tumors were grouped according to available matched TCGA clinical data on isocitrate dehydrogenase (IDH) mutation status and transcriptome molecular subtype. Figures were generated using the ggplot2 package.

### Statistics

Bars represent mean ± SEM where applicable. Statistical analyses comparing two different groups were conducted using the Student *t* test, and survival analyses were performed using the Mantel–Cox log-rank test using the Prism software package (GraphPad; RRID: SCR_002798) or R software v4.3.2 ggplot2 package. ns, not significant; *, *P* < 0.05; **, *P* < 0.01; ***, *P* < 0.001; ****, *P* < 0.0001.

### Data availability

The authors confirm that the data supporting the findings of this study are available within the article and its Supplementary Materials. The data generated in this study are available upon request to the corresponding author.

## Results

### Synthetic STING agonist MLRR does not prime the NLRP3 inflammasome to secrete IL-1β

**Figure 1:**
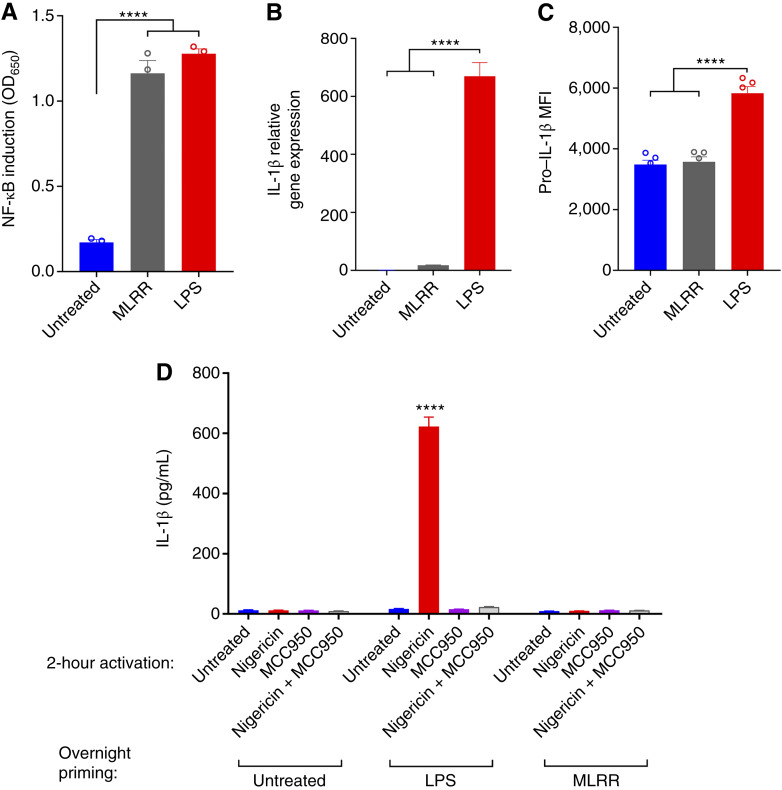
STING-induced NF-κB activation does not prime the NLRP3 inflammasome. **A,** THP1-Dual cells were incubated overnight with vehicle, MLRR (10 μg/mL), or LPS (1 μg/mL). The supernatant was harvested and NF-κB induction measured using the InvivoGen QUANTI-Blue secreted embryonic alkaline phosphatase detection system. **B** and **C,** BMDMs were incubated overnight with treatments as described in **A**. Cells were divided and processed for both IL-1β qPCR (**B**) and flow cytometry (**C**) as described in methods and stained intracellularly with eFluor780 αIL-1β proform to assess pro–IL-1β production. **D,** BMDMs were incubated overnight with priming drugs LPS or MLRR as described in **A**. Cells were then collected and treated for 2 hours in NLRP3 activator nigericin (10 µmol/L), inhibitor MCC950 (1 µmol/L), or combination as indicated. The supernatant was harvested and IL-1β secretion measured via R&D Systems murine IL-1β ELISA kit. Statistical significance was calculated using the Student *t* test. ns, not significant; ****, *P* < 0.0001. MFI, mean fluorescence intensity.

The proposed two-step process for NLRP3 inflammasome activation begins with a priming signal that induces expression of proform IL-1β via pathogen- or damage-associated molecular pattern stimulation of pattern recognition receptors such as TLR4 via LPS and subsequent NF-κB activation (16). Both LPS and the STING agonist ML-RR-S2-CDA (ADU-S100, abbreviated MLRR) induce NF-κB signaling in THP1 monocyte reporter cells (Fig. 1A). However, we found that murine BMDMs did not upregulate IL-1β gene expression (Fig. 1B) or intracellular proform IL-1β protein following MLRR treatment relative to LPS positive control by flow cytometry (Fig. 1C). Next, we treated murine BMDMs overnight for evaluation of NLRP3 priming with vehicle, LPS, or MLRR, then treated the following day for 2 hours with the NLRP3 agonist nigericin and/or inhibitor MCC950, and performed IL-1β ELISA. Although nigericin was able to induce IL-1β secretion in an NLRP3-dependent manner following LPS priming, no such secretion occurred in the untreated or MLRR priming conditions (Fig. 1D). Using the same experimental setup in the human THP1 monocyte system, we again observed that MLRR fails to prime the NLRP3 inflammasome for IL-1β secretion (Supplementary Fig. S1A). Using a Luminex system, we observed that primary human M2c macrophages do not secrete IL-1β at baseline or following cyclic dinucleotide stimulation (Supplementary Fig. S1B). These data indicate that despite signaling through the NF-κB pathway, STING activation does not serve as a priming signal for NLRP3 inflammasome-mediated IL-1β secretion.

### NLRP3 inflammasome activation restricts the proinflammatory conversion and therapeutic benefit of STING activation in subcutaneous GL261

**Figure 2:**
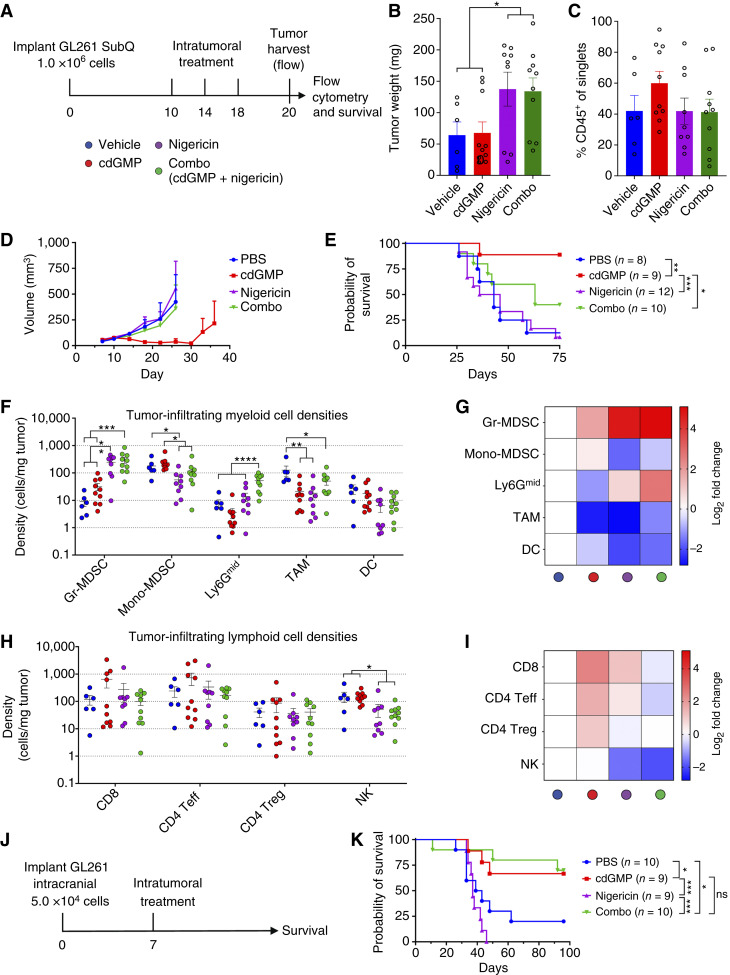
*In vivo* characterization of STING and NLRP3 inflammasome activation in GL261. **A,** Mice received subcutaneous injection of 1.0 × 10^6^ GL261 cells in 30% Matrigel and then were injected intratumorally with vehicle, STING agonist cdGMP (25 μg), NLRP3 agonist nigericin (25 μg), or combination on days 10, 14, and 18. Tumors were then processed for tumor harvest and flow cytometry as described in methods (**B, C, H–K**) or measured for survival (**D** and **E**). Data shown represent (**B**) GL261 harvested tumor weights. **C,** Analyzed CD45^+^ cell frequency of total single cells. **D,** GL261 tumor volumes and (**E**) survival data. **F–I,** Overall composition and fold changes of cell densities vs. vehicle-treated tumors for analyzed CD45^+^ (**F** and **G**) myeloid and (**H** and **I**) lymphoid cell populations. **J,** Mice received intracranial injection of 5.0 × 10^4^ GL261 cells in PBS in the right striatum via a guide bolt system, then were treated intratumorally via the guide bolt on day 7 with vehicle, STING agonist cdGMP (5 μg), NLRP3 agonist nigericin (5 μg), or combination, and survival monitored (**K**). Error bars represent mean ± SEM. Statistical significance was calculated using the Student *t* test or log-rank test. ns, not significant; *, *P* < 0.05; **, *P* < 0.01; ***, *P* < 0.001; ****, *P* < 0.0001. DC, dendritic cells; SubQ, sub-cutaneous.

NLRP3 activation has been reported to recruit immunosuppressive granulocytic myeloid-derived suppressor cells (Gr-MDSCs) to tumors in melanoma, and NLRP3 inhibition reduced tumor growth in a model of GBM (18, 22, 27). To explore the impact of NLRP3 and STING activation on the GBM tumor microenvironment, we performed intratumoral injections of subcutaneous GL261 GBM tumors with single agents and combinations of STING agonist cdGMP and NLRP3 agonist nigericin, followed by survival analysis and high parameter flow cytometric profiling of the GL261 immune infiltrate (Fig. 2A; gating strategy, Supplementary Figs. S2 and S3). On day 20, nigericin significantly increased tumor mass alone or in combination with cdGMP but did not have an impact on the proportion of CD45^+^ immune cells among total analyzed single cells (Fig. 2B and C). Although cdGMP reduced GL261 tumor growth and extended survival, this effect was antagonized when combined with nigericin (Fig. 2D and E). Conversely, addition of the NLRP3 inhibitor MCC950 had no deleterious impact on the GL261 tumor growth (Supplementary Fig. S4A) or survival benefit of cdGMP (Supplementary Fig. S4B). We next used high parameter flow cytometry to analyze the impact of STING and NLRP3 activation on the global immune composition of GL261 tumors. We found that local delivery of nigericin alone or in combination with cdGMP induced a dramatic increase in Gr-MDSC density within GL261 tumors and led to reduced densities of monocytic myeloid-derived suppressor cells (Mono-MDSC), CD11c^+^ dendritic cells, and NK cells (Fig. 2F–I). Interestingly, combination treatment led to a dramatic increase in the density of a CD11b^+^ Ly6C^−^ Ly6G^intermediate^ population that was absent in untreated and single agent tumors (Fig. 2F and G; Supplementary Fig. S2). To determine whether this therapeutic antagonism was present in the CNS, in which Gr-MDSC are sparse and neutrophil recruitment is restricted, we next performed intratumoral injection of intracranial GL261 tumors with single agents and combinations of STING agonist cdGMP and NLRP3 agonist nigericin (Fig. 2J). Mice were monitored for survival and euthanized when displaying neurologic symptoms and then necropsied to confirm intracranial tumor burden. Although in the periphery, NLRP3 activation antagonized the therapeutic benefit of STING activation, this effect was not observed in the CNS in which the combination and cdGMP alone were statistically indistinguishable (Fig. 2K).

### NLRP3 inflammasome activation expands the immunosuppressive myeloid stroma in subcutaneous GL261

**Figure 3:**
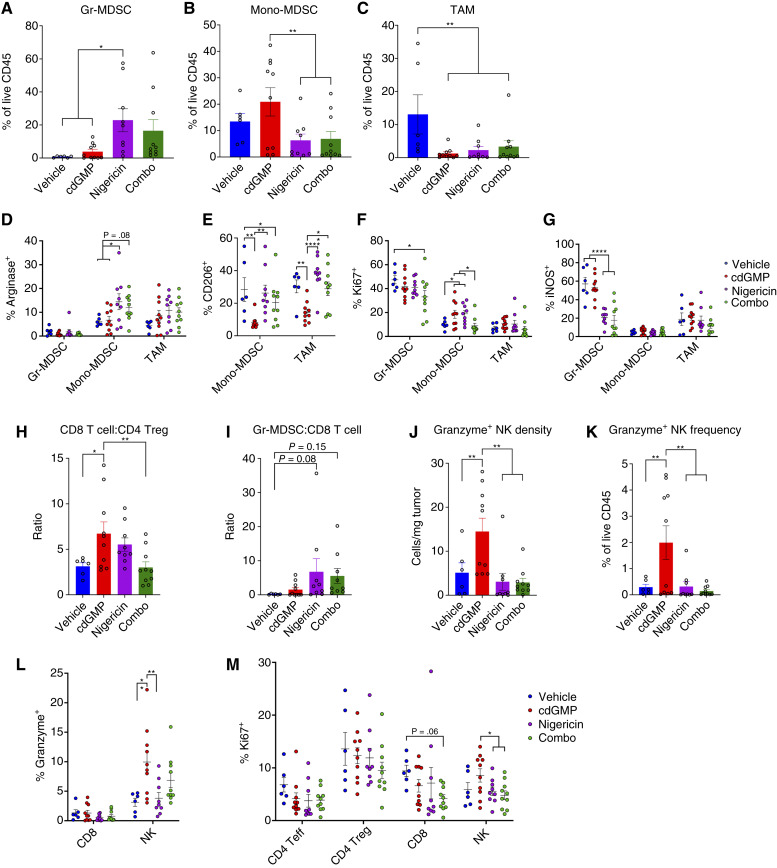
*In vivo* analysis of the GL261 tumor immune microenvironment following STING and NLRP3 inflammasome activation. Mice received subcutaneous injection of 1.0 × 10^6^ GL261 cells in 30% Matrigel and then were injected intratumorally with vehicle (*n* = 6), STING agonist cdGMP (25 μg; *n* = 10), NLRP3 agonist nigericin (25 μg; *n* = 9), or combination (*n* = 10) on days 10, 14, and 18. Tumors were then processed for tumor harvest and flow cytometry as described in methods. Data shown represent Gr-MDSC (**A**), Mono-MDSC (**B**), and TAM (**C**) frequency as a percent of total analyzed CD45^+^ cells. **D,** Arginase, (**E**) CD206, (**F**) Ki67, and (**G**) iNOS expression on Gr-MDSC, Mono-MDSC, and TAM populations as indicated. Ratios of number of (**H**) CD8 T cells/FOXP3^+^ CD4 Tregs and (**I**) Gr-MDSC/CD8 T cells. Among NK cells, granzyme^+^ NK cells were identified and reported as (**J**) density per mg of tumor tissue and (**K**) frequency as a percent of total analyzed CD45^+^ cells. **L,** Granzyme and (**M**) Ki67 expression on CD4, CD8, and NK cell populations as indicated. Error bars represent mean ± SEM. Statistical significance was calculated using the Student *t* test. ns, not significant; *, *P* < 0.05; **, *P* < 0.01; ****, *P* < 0.0001. iNOS, inducible nitric oxide synthase.

As NLRP3 activation antagonized the therapeutic effect of cdGMP and increased the density of Gr-MDSCs in subcutaneous GL261 tumors, we next sought to profile the phenotypes of the myeloid infiltrate in cdGMP- and nigericin-treated GL261 tumors (Fig. 2A; gating strategy, Supplementary Figs. S2 and S3). Nigericin increased the frequency of Gr-MDSCs among total CD45^+^ immune cells alone or in combination with cdGMP (Fig. 3A). Local cdGMP treatment caused an expansion of Mono-MDSCs; however, they may more closely functionally resemble inflammatory myeloid cells reported to infiltrate other STING-treated tumor types. This Mono-MDSC expansion was reversed by nigericin (Fig. 3B). Both cdGMP and nigericin reduced the frequency of TAMs in the CD45^+^ immune infiltrate (Fig. 3C). Among both Mono-MDSCs and TAMs, nigericin induced immunosuppressive polarization alone and in combination with cdGMP, including an increase in arginase expression and a reversal of cdGMP-induced reduction of the CD206 marker of M2 polarization (Fig. 3D and E). Combination treatment lowered expression of the proliferation marker Ki67 across the analyzed myeloid infiltrate (Fig. 3F). Finally, nigericin both alone and in combination with cdGMP reduced the expression of the proinflammatory enzyme inducible nitric oxide synthase in Gr-MDSCs and TAMs (Fig. 3G).

### STING activation–driven CD8 T- and NK-cell proinflammatory effects are restricted by NLRP3 inflammasome activation in subcutaneous GL261

To identify NLRP3 activation effects on possible cytotoxic effectors of cdGMP-induced antitumor immunity, we next sought to profile the phenotypes of the lymphoid infiltrate in cdGMP- and nigericin-treated subcutaneous GL261 tumors (Fig. 2A, gating strategy, Supplementary Figs. S2 and S3). Local cdGMP treatment increased the therapeutic ratio of CD8 Teff cells to CD4 Tregs, which was reversed with the addition of nigericin (Fig. 3H). Nigericin also trended toward increasing the ratio of Gr-MDSCs to CD8 Teff cells alone and in combination with cdGMP (Fig. 3I). Notably, cdGMP dramatically increased the density, as well as frequency among CD45^+^ immune cells, of granzyme B^+^ cytotoxic NK cells, which was completely reversed with the addition of nigericin (Fig. 3J and K). Granzyme B expression was higher among NK cells than CD8 T cells across treatment conditions. cdGMP further increased NK granzyme B expression, which was partially reversed with the addition of nigericin (Figure 3L). Finally, nigericin treatment reduced Ki67 expression on CD4 Teff and CD8 Teff cells, which was partially reversed by combination with cdGMP (Fig. 3M).

### NLRP3 activation expands the immunosuppressive myeloid stroma in intracranial GL261

**Figure 4:**
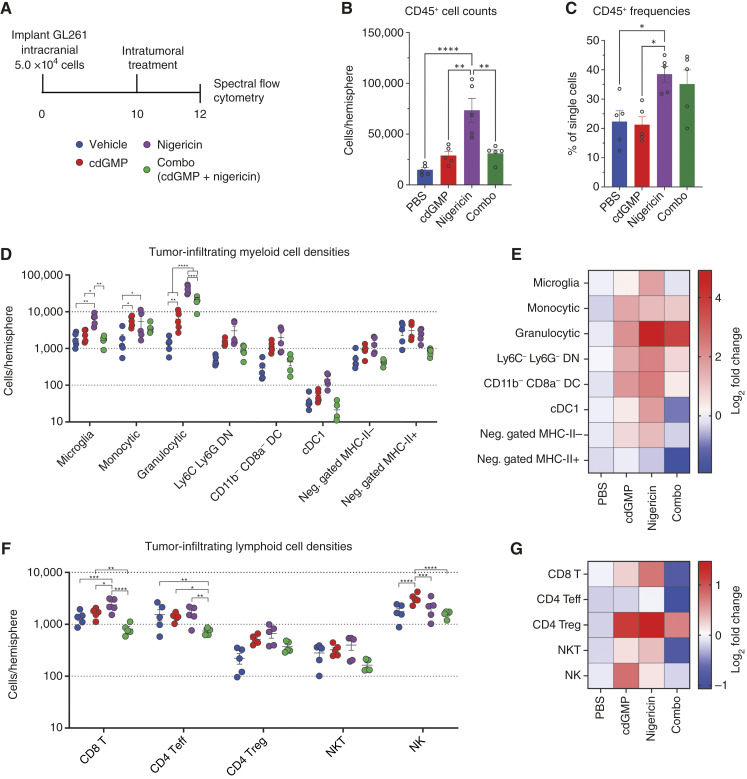
*In vivo* analysis of intracranial GL261 tumor immune microenvironment composition following STING and NLRP3 inflammasome activation. **A,** Mice received intracranial injection of 5.0 × 10^4^ GL261 cells, then were treated with 5 μg of cdGMP and/or nigericin, and tumor-bearing hemispheres harvested 48 hours following treatment for spectral flow cytometry analysis. **B,** Live CD45^+^ cell densities reported as cells per tumor-bearing hemisphere. **C,** Live CD45^+^ cell frequency as a fraction of total single cells. **D–G,** Overall composition and fold changes of cell densities vs. vehicle-treated tumors for analyzed CD45^+^ (**D** and **E**) myeloid and (**F** and **G**) lymphoid cell populations. Error bars represent mean ± SEM. Statistical significance was calculated using the Student *t* test. ns, not significant; *, *P* < 0.05; **, *P* < 0.01; ***, *P* < 0.001; ****, *P* < 0.0001. cDC1, type 1 conventional dendritic cells; Neg., negatively.

**Figure 5:**
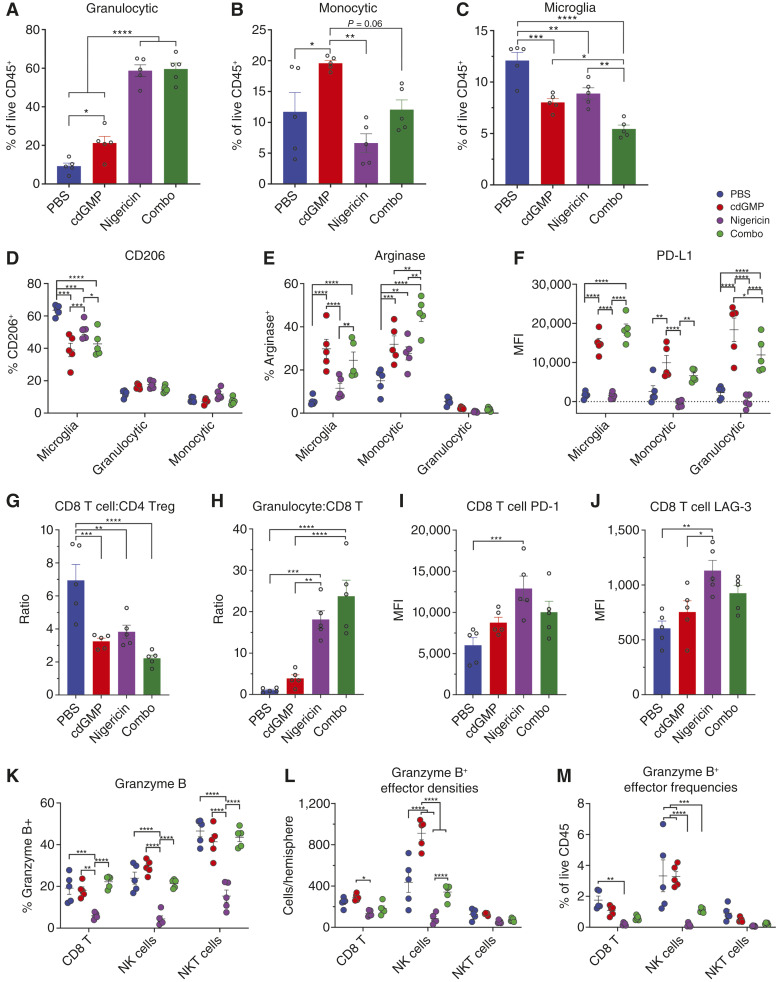
*In vivo* analysis of intracranial GL261 tumor immune microenvironment phenotypes following STING and NLRP3 inflammasome activation. Mice received intracranial injection of 5.0 × 10^4^ GL261 cells, then were treated with 5 μg of cdGMP and/or nigericin, and tumor-bearing hemispheres harvested 48 hours following treatment for spectral flow cytometry analysis. **A,** Gr-MDSC, (**B**) Mono-MDSC, and (**C**) microglia frequency as a percent of total analyzed CD45^+^ cells. **D,** CD206, (**E**) arginase, and (**F**) PD-L1 expression on Gr-MDSCs, Mono-MDSCs, and microglia as indicated. Ratios of the number of (**G**) CD8 T cells/FOXP3^+^ CD4 Tregs and (**H**) Gr-MDSC/CD8 T cells. Expression of (**I**) PD-1 and (**J**) LAG-3 on CD8 T cells. **K,** Granzyme expression on CD8 Teff, NK cells, and NKT cells as indicated. Among CD8 Teff, NK cells, and NKT cells, granzyme^+^ cells were identified and reported as (**L**) density as cells per tumor-bearing hemisphere and (**M**) frequency as a percent of total analyzed CD45^+^ cells. Error bars represent mean ± SEM. Statistical significance was calculated using the Student *t* test. ns, not significant; *, *P* < 0.05; **, *P* < 0.01; ***, *P* < 0.001; ****, *P* < 0.0001. MFI, mean fluorescence intensity.

Because NLRP3 activation did not antagonize the therapeutic benefit of STING activation in the intracranial setting, we sought to determine whether similar expansion of immunosuppressive myeloid populations and restriction of cdGMP-mediated proinflammatory conversion occur in GL261 in the CNS. We implanted intracranial GL261 and treated with a single intratumoral dose of cdGMP, nigericin, or combination and harvested tumor-bearing hemispheres 48 hours after treatment for spectral flow cytometry analysis (Fig. 4A; gating strategy, Supplementary Figs. S5 and S6). We found that nigericin significantly increased CD45^+^ immune cell densities and frequencies in intracranial GL261-bearing hemispheres, largely due to expanded Gr-MDSCs as well as microglia and Mono-MDSCs (Fig. 4B–E). Unlike subcutaneous GL261, we also found that intracranial nigericin expanded tumor-infiltrating Tregs. Although nigericin alone was insufficient to reduce NK-cell infiltration, the addition of nigericin was sufficient to reverse the cdGMP-mediated increase in NK-cell infiltration (Fig. 4F and G). In both nigericin- and combination-treated tumors, Gr-MDSCs constituted almost 60% of all live CD45^+^ cells, over triple the fraction observed in subcutaneous GL261 tumors (Fig. 5A). Intracranial cdGMP expanded the fraction of Mono-MDSCs; however, this effect was reversed when combined with nigericin (Fig. 5B). Although the density of microglia increased with nigericin monotherapy (Fig. 4D), their frequency within the CD45^+^ immune infiltrate decreased across all treatment conditions (Fig. 5C). STING activation reduced microglial CD206 expression; however, unlike macrophages and monocytes in the subcutaneous setting, this effect was not reversed with the addition of nigericin (Fig. 5D). Consistent with previous reports of STING activation in GBM, cdGMP elevated microglial arginase expression. Conversely, nigericin neither affected microglial arginase expression as a monotherapy or in combination with cdGMP (Fig. 5E). A similar pattern was observed across microglia, Mono-MDSCs, and Gr-MDSC PD-L1, in which cdGMP significantly elevated PD-L1 expression both alone and in combination with nigericin (Fig. 5F).

### NLRP3 activation partially reverses STING activation–driven proinflammatory CD8 T- and NK-cell effects in intracranial GL261

To identify NLRP3 activation effects on possible cytotoxic effectors of cdGMP-induced antitumor immunity, we next sought to profile the phenotypes of the lymphoid infiltrate in cdGMP- and nigericin-treated intracranial GL261 tumors (Fig. 4A; gating strategy, Supplementary Figs. S5 and S6). The ratios of CD8 T cells to CD4 Tregs decreased across all treatment conditions, contradicting the elevated ratio observed in cdGMP-treated subcutaneous GL261 (Fig. 5G). Nigericin dramatically increased the ratio of Gr-MDSCs to CD8 Teff cells, both alone and in combination with cdGMP (Fig. 5H). Consistent with the observed expansion of suppressive myeloid cells, CD8 T cells in nigericin-treated tumors increased expression of the exhaustion markers PD-1 and LAG-3 (Fig. 5I and J). We again observed that nigericin reduces the fraction of granzyme^+^ CD8 Teff and NK cells as well as NKT cells. However, contradictory to our observations in subcutaneous GL261, this nigericin-induced reduction in granzyme expression was not maintained in combination-treated intracranial GL261 tumors (Fig. 5K). Intracranial cdGMP expanded both the density per tumor-bearing hemisphere and frequency as a fraction of total CD45^+^ immune cells of cytotoxic granzyme^+^ NK cells; however, this effect was reversed with the addition of nigericin and the resulting large influx of Gr-MDSCs (Fig. 5L and M). Expression of the proliferation marker Ki67 across myeloid (Supplementary Fig. S7A) and lymphoid lineages (Supplementary Fig. S7B) in treated GL261 intracranial tumors also failed to show any benefit to combination therapy.

### Neutrophil gene score predicts survival and varies across molecular subtypes in patients with primary GBM

**Figure 6:**
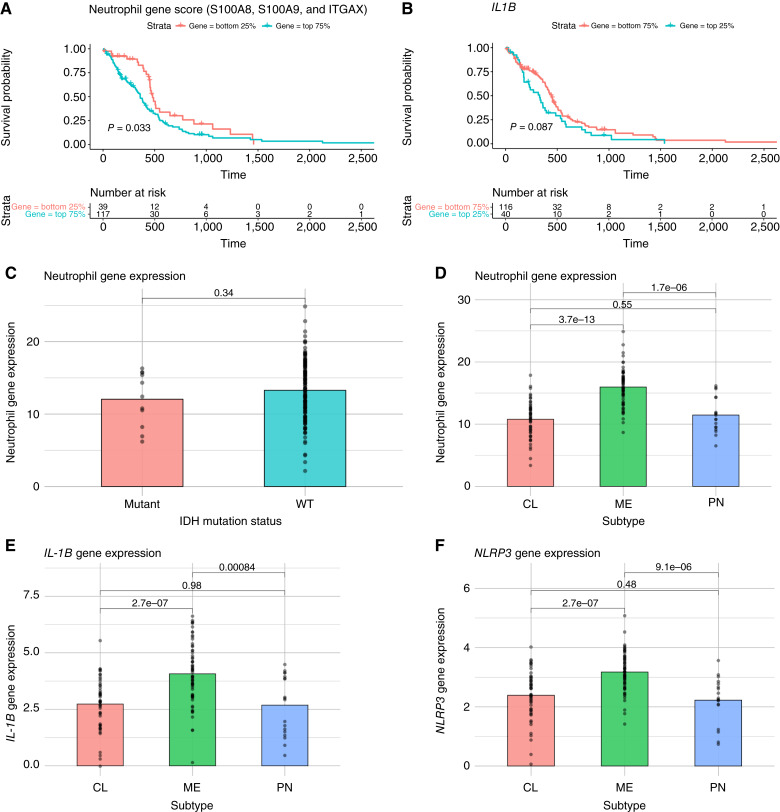
TCGA analysis of neutrophil-related genes in patients with primary GBM. Bulk RNA-seq transcriptome profiling data were normalized, and neutrophil gene scores based on the expression of S100A8, S100A9, and ITGAX were assigned to each patient sample. **A,** Survival was analyzed comparing patients with low (bottom quartile) and high (top 75%) neutrophil gene scores. **B,** Survival was analyzed comparing patients with high (top quartile) and low (bottom 75%) expression of the gene *IL1B*. Neutrophil gene scores were compared across IDH mutation status (**C**) and transcriptome molecular subtypes (**D**). Expression of *IL1B* (**E**) and *NLRP3* (**F**) genes were compared across transcriptome molecular subtypes. Error bars represent mean ± SEM. Statistical significance was calculated using the Student *t* test or log-rank test. CL, classical; ME, mesenchymal; PN, proneural; WT, wild-type.

As the detrimental survival effects of nigericin in the periphery were not reflected in intracranial GL261 GBM, we next sought to determine whether subsets of patients with GBM with higher tumor neutrophil densities may be present. Neutrophil gene scores were calculated based on normalized expression of S100A8, S100A9, and ITGAX (26) among patients with primary GBM in the TCGA transcriptome profiling database. Patients with low neutrophil gene scores had extended survival compared with those with high scores (Fig. 6A). Additionally, patients with high expression of the *IL1B* gene encoding pro–IL-1β trended toward lower survival than those with low *IL1B* expression (Fig. 6B). As differential infiltration of granulocytes has been observed in syngeneic RCAS/tva murine glioma models differing only in the presence of wild-type or mutant IDH1 (28), we sought to determine whether this was reflected in human GBM samples. Unlike in the murine RCAS/tva system, neutrophil gene scores did not significantly differ between patients with IDH wild-type and mutant tumors (Fig. 6C). To determine whether neutrophil gene expression differed between molecular subtypes, we divided patients by TCGA transcriptomic molecular GBM subtypes and compared neutrophil gene scores. Neutrophil gene scores were significantly higher among mesenchymal tumors compared with those with classical and proneural subtypes (Fig. 6D). Additionally, expression of the genes *IL1B* (Fig. 6E) and *NLRP3* (Fig. 6F) was significantly elevated in mesenchymal subtype tumors.

## Discussion

Several studies have linked the cGAS-STING and NLRP3 inflammasome pathways, including by demonstrating that STING activation can activate LPS-primed NLRP3 inflammasomes, protect the NLRP3 complex from ubiquitination, and even prime some NLRP3 inflammasome components (15, 29). One study has reported that the canonical cGAS product 2′3′cGAMP can prime NLRP3 and caspase-1 transcription at levels comparable with classical LPS-TLR4 NLRP3 priming. This study also showed that 2′3′cGAMP can weakly induce IL-1β transcription; however, LPS treatment induced IL-1β transcription at about 20-fold higher level than observed with 2′3′cGAMP (15). In our system, using the synthetic STING agonist MLRR, which has about 10-fold higher potency than 2′3′cGAMP (30), no priming of the NLRP3 inflammasome was observed via either intracellular pro–IL-1β protein expression or IL-1β secretion following treatment with the NLRP3 complex agonist nigericin. At the mRNA level, MLRR did very slightly increase pro–IL-1β transcription, albeit at about 40-fold lower level than LPS. This raises questions about the mechanism of 2′3′cGAMP-induced NLRP3 priming and activation and whether the potential for STING activation to orchestrate NLRP3-induced protumorigenic effects is clinically relevant.

Consistent with reports that NLRP3 activation drives dense Gr-MDSC infiltration in melanoma (18), we observed that local nigericin treatment led to a considerable increase in both the density and the frequency among immune cells of Gr-MDSCs in peripheral subcutaneous GL261 tumors. This may help to connect our understanding of NLRP3-driven immunosuppressive tumor immunology in other non-CNS cancers to the observations in GBM that NLRP3 inhibition slows tumor growth and synergizes with irradiation (22). Considering that NLRP3 inflammasome activity has been observed in human GBM tissue samples, this observation may also suggest NLRP3 inhibitors of current clinical interest has to be tested in GBM as well (21), particularly in patients with the mesenchymal molecular tumor subtype, which we observed to have elevated neutrophil gene signatures.

Our findings also demonstrate that the STING agonist cdGMP can cure GBM tumors and induce proinflammatory conversion of the tumor immune microenvironment, consistent with other studies on STING agonism in GBM (8, 9). Notably, concurrent NLRP3 activation strongly antagonized the therapeutic efficacy of cdGMP in peripheral subcutaneous GL261 but not in CNS orthotopic GL261. Several cdGMP-induced proinflammatory effects, including reversal of M2 TAM polarization, improved CD8 Teff to CD4 Treg ratios, and dramatically increased densities of cytotoxic granzyme B^+^ NK cells, were entirely reversed with concurrent nigericin treatment in the subcutaneous setting. Interestingly, NLRP3 signaling has been shown to suppress NK cell antitumor immunity in a methylcholanthrene sarcoma model (31). Subcutaneous cdGMP also failed to reverse several immunosuppressive effects observed following nigericin treatment, including dense Gr-MDSC infiltration, reduced Gr-MDSC inducible nitric oxide synthase expression, and increased Mono-MDSC arginase expression. Previous studies from our group have shown that Gr-MDSCs represent less than one percent of the total CD45^+^ immune infiltrate in intracranial GL261 tumors at baseline, as well as following either PD-1 or CTLA-4 systemic blockade (25). Although a large reservoir of neutrophils is maintained in the periphery compared with their restricted infiltration into brain tumors (4, 5, 25, 32, 33), we nevertheless found that intracranial nigericin induced a dramatic increase in Gr-MDSC infiltration in GL261 tumor-bearing hemispheres. Previous studies have identified infiltrating inflammatory monocytic lineage cells infiltrating STING agonist-treated tumors both in the periphery and in the CNS. Notably, we found that nigericin reversed the STING-mediated myeloid CD206 expression reduction in subcutaneous, but not intracranial, GL261. This may suggest that NLRP3 activation restricts STING activation-induced inflammatory myeloid cell infiltration and polarization in the periphery, but not the CNS, which may contribute to the differential survival outcomes when combined in the subcutaneous versus intracranial settings. Additionally, although nigericin monotherapy reduced NK-cell granzyme expression in the CNS setting, NK cells in combination-treated tumors retained elevated granzyme expression. As previous studies have identified cytotoxic NK cells as necessary for the therapeutic efficacy in the intracranial GL261 tumor model, the failure of nigericin to reduce NK granzyme expression when combined with cdGMP, specifically in the CNS but not subcutaneous setting, may also contribute to the observed differential survival outcomes.

Notably, patients with low neutrophil gene signatures demonstrated extended survival compared with those with high gene signatures, suggesting a potential role for neutrophil infiltration on disease outcome in GBM. This is consistent with a previous study that determined that TCGA patients with GBM with higher tumor-associated neutrophil content (computed via CIBERSORTx) had poorer survival outcomes than patients with lower tumor-associated neutrophil content (34). In this context, it is notable that neutrophil gene signatures vary across molecular subtypes. Indeed, mesenchymal subtype tumors are significantly enriched for neutrophil gene signatures as well as gene expression of both IL1B and NLRP3. Although the potential for NLRP3-mediated neutrophil infiltration and orchestration of protumorigenic effects may be limited in classical and proneural tumors, it may be clinically relevant in mesenchymal tumors that may have elevated neutrophil infiltration and NLRP3 inflammasome activity.

In summary, these data suggest that NLRP3 activation can limit the therapeutic efficacy and proinflammatory effects of STING agonism in GBM. Again, because NLRP3 activity has been observed in human and murine GBM tissue samples and because NLRP3 activity restricts STING agonist therapeutic efficacy, our data suggest that combining STING activation and NLRP3 inhibition in GBM tumors with NLRP3 activity may provide therapeutic synergy and merits further investigation, particularly in mesenchymal and neural GBM tumors with elevated neutrophil infiltration and inflammasome activity.

## Supplementary Material

Supplementary Figure 1 Supplementary Figure 1

Supplementary Figure 2 Supplementary Figure 2

Supplementary Figure 3 Supplementary Figure 3

Supplementary Figure 4 Supplementary Figure 4

Supplementary Figure 5 Supplementary Figure 5

Supplementary Figure 6 Supplementary Figure 6

Supplementary Figure 7 Supplementary Figure 7
