# Supplementary material for: NLRP3 Inflammasome Activation Expands the Immunosuppressive Myeloid Stroma and Antagonizes the Therapeutic Benefit of STING Activation in Glioblastoma
Source: Cancer Res Commun. 2025 Jun 13;5(6):960–72. doi: 10.1158/2767-9764.CRC-23-0189 (PMC12163576; doi:10.1158/2767-9764.CRC-23-0189)
Supplement: Supplementary Figure 1 [file crc-23-0189_supplementary_figure_1_suppsf1.pdf]

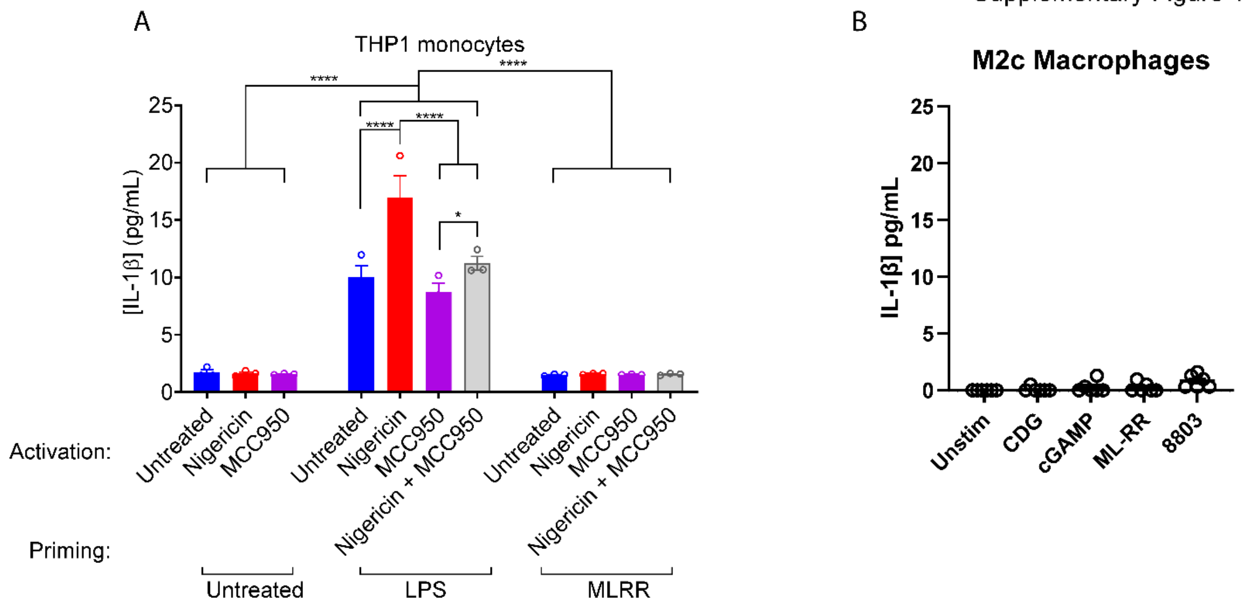

Supplementary Figure 1: (A) THP-1 Dual cells were incubated overnight with vehicle, MLRR (10 µg/mL), or LPS (1 µg/mL). Cells were then collected and treated for two hours in NLRP3 activator Nigericin (10 µM), inhibitor MCC950 (1 µM) or combination as indicated. Supernatant was harvest and IL-1β secretion measured via Thermo Scientific mouse IL-1β ELISA kit. **(B)** Human M2c macrophages were differentiated from PBMC monocytes as described in Methods, then stimulated by indicated CDN at 10 µg/mL in the presence of supportive recombinant M-CSF for 72 hours. Secreted analyte concentrations in culture supernatants measured using Luminex multiplex cytokine/chemokine analysis. Data are inclusive of macrophages from six unique donors in two independent batches. Error bars represent mean plus or minus standard error of the mean. Statistical significance was calculated using Student's t-test. ns, not significant; *P< 0.05, **P < 0.01, ***P < 0.001, ****P < 0.0001.
