# Supplementary material for: NLRP3 Inflammasome Activation Expands the Immunosuppressive Myeloid Stroma and Antagonizes the Therapeutic Benefit of STING Activation in Glioblastoma
Source: Cancer Res Commun. 2025 Jun 13;5(6):960–72. doi: 10.1158/2767-9764.CRC-23-0189 (PMC12163576; doi:10.1158/2767-9764.CRC-23-0189)
Supplement: Supplementary Figure 2 [file crc-23-0189_supplementary_figure_2_suppsf2.pdf]

Supplementary Figure 2

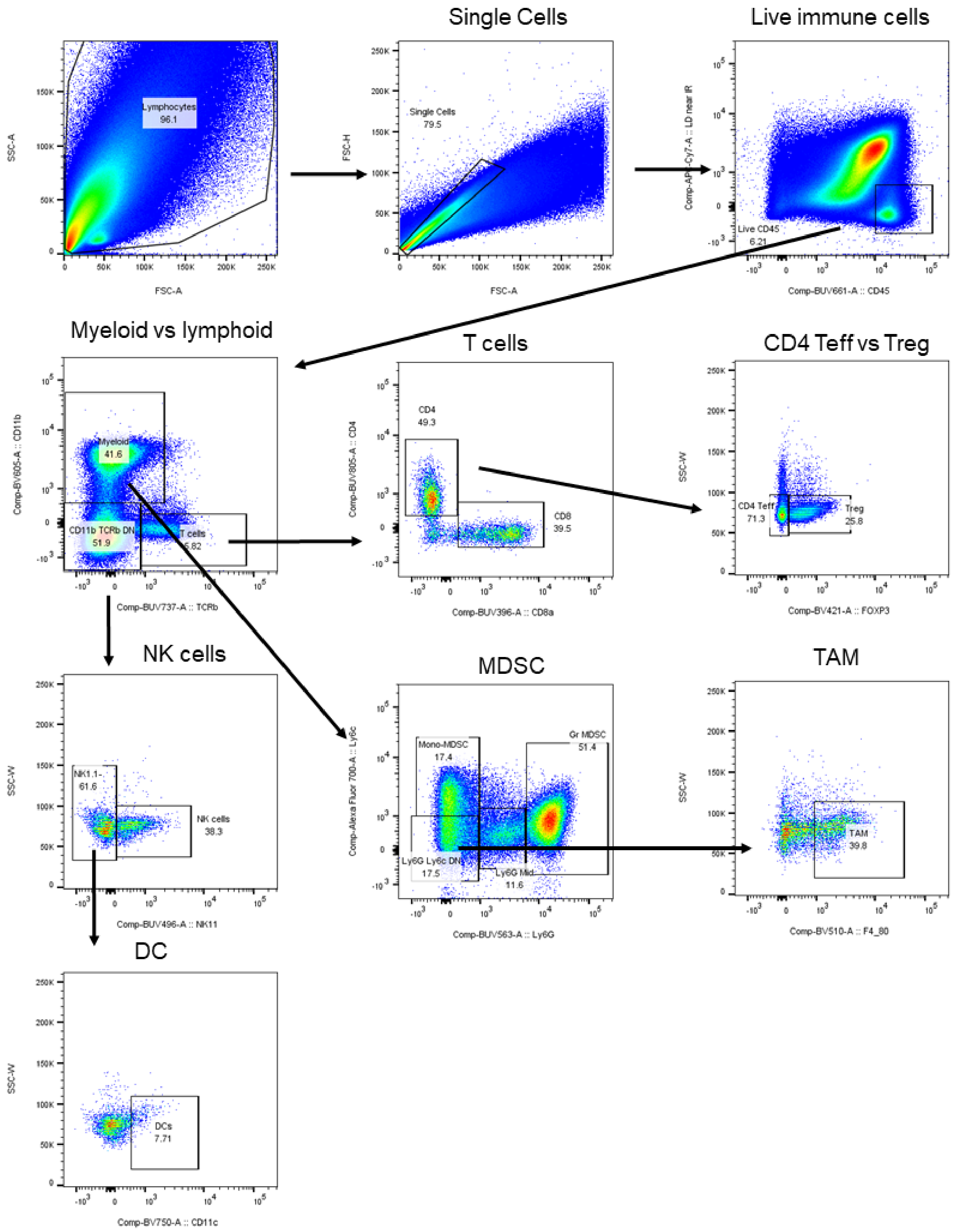

Supplementary Figure 2: Population gating strategy for GL261 tumor immune infiltrate analysis.
