# Supplementary material for: NLRP3 Inflammasome Activation Expands the Immunosuppressive Myeloid Stroma and Antagonizes the Therapeutic Benefit of STING Activation in Glioblastoma
Source: Cancer Res Commun. 2025 Jun 13;5(6):960–72. doi: 10.1158/2767-9764.CRC-23-0189 (PMC12163576; doi:10.1158/2767-9764.CRC-23-0189)
Supplement: Supplementary Figure 4 [file crc-23-0189_supplementary_figure_4_suppsf4.pdf]

A

Supplementary Figure 4

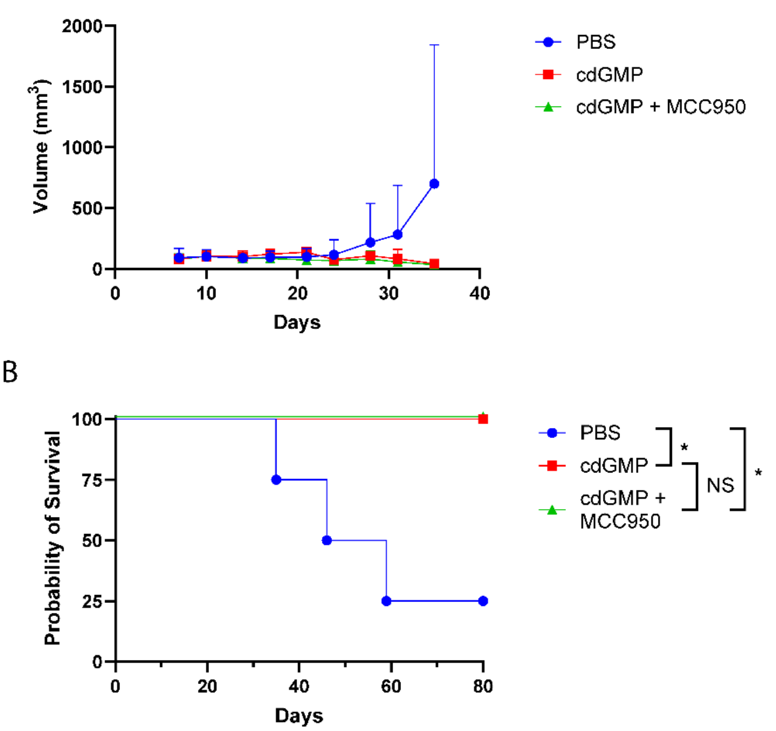

Supplementary Figure 4: Mice received subcutaneous injection of 1.0×10^6 GL261 cells in 30% Matrigel, then were injected intratumorally with vehicle, STING agonist cdGMP (25 µg), NLRP3 inhibitor MCC950 (25 µg) combined with cdGMP on days 10, 14, and 18. Tumors were then measured for **(A)** tumor volume and **(B)** survival.
