# Supplementary material for: NLRP3 Inflammasome Activation Expands the Immunosuppressive Myeloid Stroma and Antagonizes the Therapeutic Benefit of STING Activation in Glioblastoma
Source: Cancer Res Commun. 2025 Jun 13;5(6):960–72. doi: 10.1158/2767-9764.CRC-23-0189 (PMC12163576; doi:10.1158/2767-9764.CRC-23-0189)
Supplement: Supplementary Figure 7 [file crc-23-0189_supplementary_figure_7_suppsf7.pdf]

Supplementary Figure 7

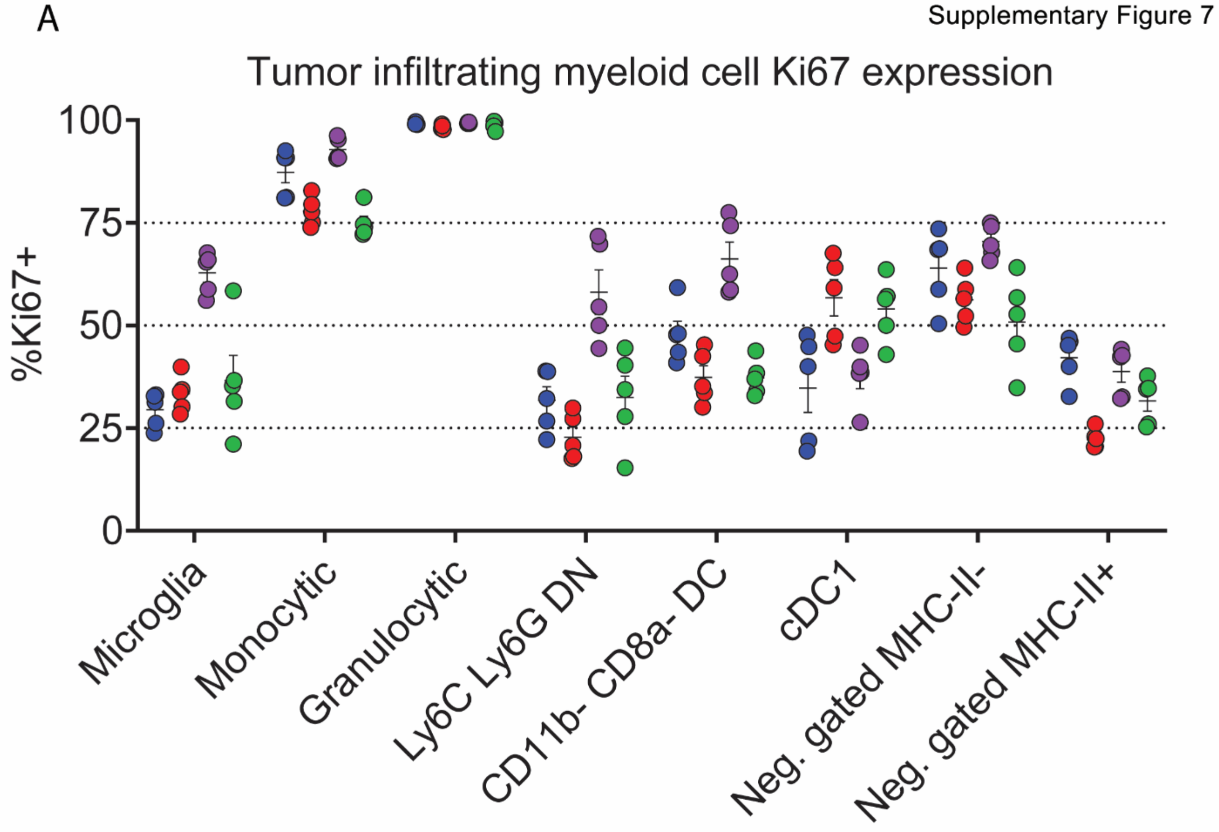

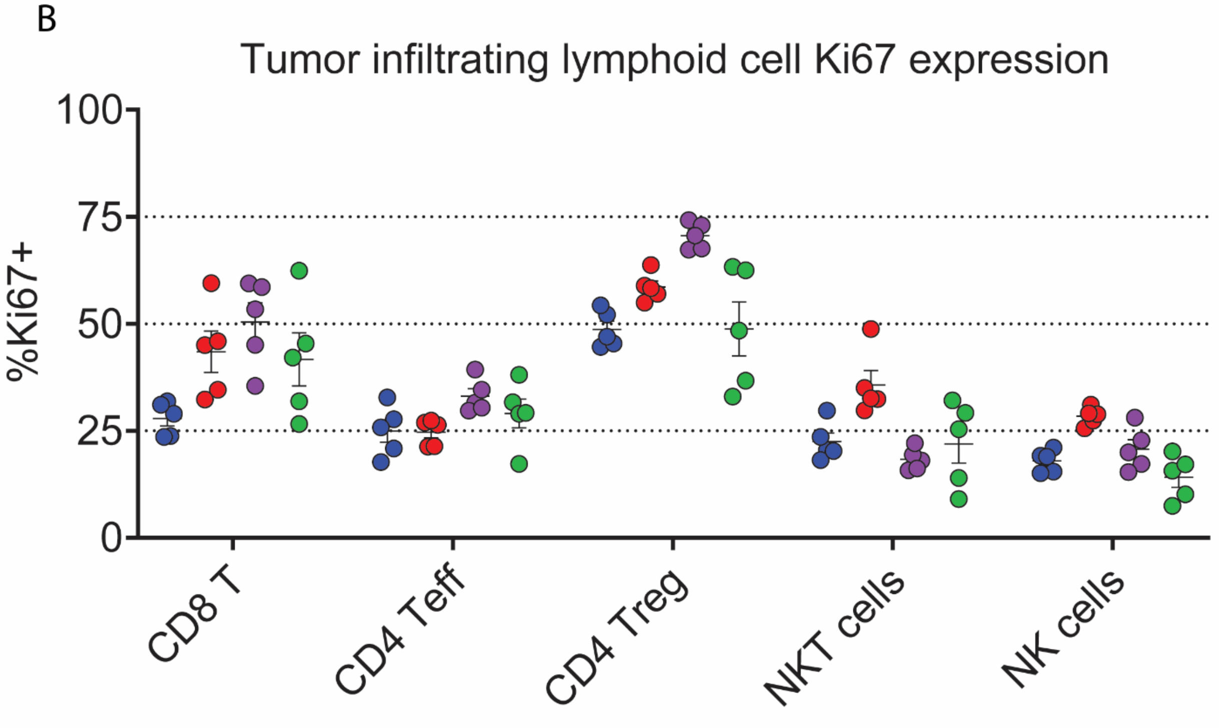

Supplementary Figure 7: A) Myeloid and **B**) lymphocyte proliferation (Ki67) from Nigericin treated intra-cranial GL261 tumors.
